# Stem Cell for Cancer Immunotherapy: Current Approaches and Challenges

**DOI:** 10.1007/s12015-025-10933-5

**Published:** 2025-07-12

**Authors:** Zainab Alali, Umme Tamanna Ferdous, Alexis Nzila, Farhana Easmin, Adnan Shakoor, Abdul Wasy Zia, Shihab Uddin

**Affiliations:** 1 Department of Bioengineering, King Fahd University of Petroleum & Minerals, 31261 Dhahran, Saudi Arabia; 2 Center for Biosystems and Machines, King Fahd University of Petroleum & Minerals, 31261 Dhahran, Saudi Arabia; 3 Interdisciplinary Research Center for Membranes and Water Security, King Fahd University of Petroleum and Minerals, 31261 Dhahran, Saudi Arabia; 4 Department of Biological Engineering, Koch Institute for Integrative Cancer Research, Massachusetts Institute of Technology, 77 Massachusetts Avenue, Cambridge, MA 02139 USA; 5 Department of Control & Instrumentation Engineering, King Fahd University of Petroleum & Minerals, 31261 Dhahran, Saudi Arabia; 6 Institute of Mechanical, Process, and Energy Engineering (IMPEE), School of Engineering and Physical Sciences, Heriot-Watt University, Edinburgh, EH14 4AS UK

**Keywords:** Cancer, Immunotherapy, Stem cells, Microenvironment, CRISPR, CAR-T cells

## Abstract

Stem cell-based immunotherapy represents a groundbreaking advancement in cancer treatment, leveraging the immune system's inherent capacity to target and eradicate cancer cells. This review explores some of the examples of stem cells used in cancer immunotherapy, including hematopoietic, mesenchymal, and induced pluripotent stem cells (IPSCs). It also describes stem cell functionalities like modifying tumor microenvironment (TME) and developing engineered immune cells like chimeric antigen receptor (CAR)-T cells and natural killer (NK) cells. Additionally, the clinical applications of stem cells for improving cancer immunotherapies and delivering drugs directly to solid tumors are discussed. However, several challenges limit the effectiveness of stem cell technology, including safety risks, tumor avoidance by the immune system, and regulatory protocols as well as manufacturing barriers. This article reviews current advancements to overcome these challenges, such as CRISPR-based gene editing and targeted drug delivery systems and provides an outlook on emerging trends, such as the progress of personalized stem cell therapies and the increasing effectiveness of treatment by combining them with other cancer treatments.

**Graphical Abstract:**

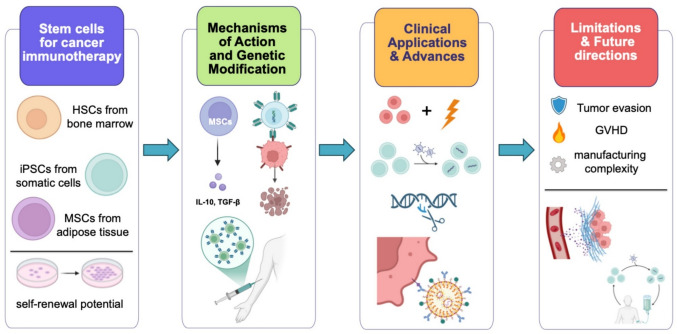

## Highlights

Stem cells are significant in cancer immunotherapy due to their tumor-targeting abilities and role in altering the tumor microenvironment.

Stem cells can modify the tumor microenvironment by delivering therapeutic agents and suppressing cancer-promoting factors.

Stem cells function as carriers for oncolytic viruses, permitting targeted elimination of cancer cells with minimal influence on healthy tissues.

The integration of CRISPR-Cas9 technology has enhanced the precision and safety of stem cell-based immunotherapies.

Personalized stem cell therapies are being considered to improve accessibility and scalability.

## Introduction

Cancer, a rapidly progressing disease of uncontrolled cellular growth, is one of the leading causes of death around the world [1]. Statistics have projected that there would be nearly 2,001,140 new cancer incidents and 611,720 cancer mortalities expected for the year 2024 in the United States, based on incidence statistics gathered from central cancer registries and mortality data collected from the National Center for Health Statistics. The same study indicates that cancer mortality rates continue to decrease because of some factors, including reduced smoking, early detection, and improved treatment options [2]. These statistics are crucial for devising and implementing strategies to reduce mortality rates through effective treatments and early cancer detection. A study indicates that approximately one-quarter of the population will develop cancer at some point during their lifetimes [3]. The overall lifetime risk of cancer is slightly greater for men than for women. Not only this, but also the lifetime risk of developing cancer varies significantly between regions, with the overall risk being higher in high-income countries due to longer life expectancies and higher detection rates [3]. The Global Cancer Observatory estimates the approximate number of incidences and mortality of cancer worldwide in both males and females of all ages from 2022 to 2045. The data indicate an increase in both cancer incidence and mortality over time for both females and males. However, cancer mortality rates are lower than incidence rates [4]. According to the Global Cancer Observatory, in 2022, the global impact of cancer was profound, with around 20 million new cases diagnosed and 9.7 million lives lost to the disease. These numbers highlight the critical need for ongoing research, enhanced prevention efforts, and greater access to high-quality cancer care for everyone, regardless of where they live. Another study expects that by 2040, breast cancer, melanoma, lung cancer, colorectal cancer, and prostate cancer will be the most common cancers in the United States. The same study estimates that lung cancer (63,000 deaths) will continue to be the leading cause of tumor mortality through 2040, with pancreatic cancer (46,000 deaths) and liver and intrahepatic bile duct cancer (41,000 deaths) overtaking colorectal cancer (34,000 deaths) to become the second and third most common causes of cancer-related death, respectively [5].

Cancer arises from mutations or alterations in genes that regulate cell activity and control how cells develop and divide, including proto-oncogenes, tumor suppressor genes, and DNA repair genes [6–8]. These genetic modifications lead to uncontrolled cellular growth and reproduction. Several approaches to controlling and treating cancer include traditional and advanced treatments. Sometimes, conventional cancer treatments have some limitations and might lead to incomplete tumor eradication and relapse. The most commonly used conventional treatments include chemotherapy, surgery, and radiotherapy. Advanced cancer treatments involve immunotherapy, targeted therapy, gene therapy, and photodynamic therapy.

Immunotherapy, a novel approach, targets and destroys cancer cells by stimulating the immune system. This transformative therapeutic modality holds the potential to promote long-term tumor regression and enhance overall survival across various cancer types [9]. Over the past ten years, immunotherapy has given patients with varying kinds of advanced cancer remarkable clinical outcomes [10]. A variety of experiments and clinical research have demonstrated that immunotherapy offers distinctive advantages over conventional cancer therapies, leading to longer overall survival (OS) and prolonged progression-free survival (PFS) [11]. Stem cells are undifferentiated cells with the ability to differentiate into a multitude of cell types, including NK, dendritic, and T cells. The employment of stem cells in cancer therapy is gaining recognition among researchers as a promising translational treatment option, largely due to the distinctive characteristics inherent to these cells. These include anti-cancer properties, the capacity to differentiate into various cell types, self-renewal, and the ability to home to tumor sites [12, 13]. Some types of stem cells, such as the mesenchymal (MSCs) and hematopoietic stem cells (HSCs), have a significant role in enhancing cancer immunotherapy because of their capability to modulate the immune response and deliver therapies directly to tumors.

Moreover, T cells are an example of stem cells that have demonstrated a major part in cancer immunotherapy since their discovery, leading to novel treatments such as CAR-T cell therapy [14]. On the other hand, NK cells represent a distinctive ability to directly identify and eliminate tumor cells without needing antigen presentation. This property distinguishes them from other adaptive immune cells, such as T and B cells [15]. Using stem cell-based immunotherapy to overcome the limitations of the current therapeutic modalities is a promising approach. Genetically modified stem cells can express specific genes, such as cytokines, chemokines, and CARs, to enhance their anti-tumor efficacy [16]. Introducing CAR into T cells through CRISPR-Cas9 gene editing enables them to recognize and destroy cancer cells [16]. Along with addressing the obstacles to their application in cancer treatment, this review focuses at and evaluates the most recent developments in stem cell-based immunotherapy.

## Stem Cells Used in Cancer Immunotherapy

Stem cells are undifferentiated cells with an ability to develop into cell types that are more specialized. They are capable of renewing themselves through cell division. Stem cells are derived into a variety of forms, each with unique properties, including their ability to renew themselves, their unspecialized nature, and their capacity for differentiation [17]. Because of their ability to specialize in diverse cell types and self-renew, stem cells have considerable potential in cancer therapy and regenerative medicine, as they can treat many diseases. Engineered stem cells can enhance treatment efficiency and deliver drugs directly to the affected area [18, 19]. T cells and NK cells can be created from stem cells to target and eliminate cancer [20]. Stem cells can be found in both embryos and adult cells [21]. Stem cells can be classified based on their differentiation potency or source of origin. Table 1 summarizes the applications, advantages, advances, and challenges of different types of stem cells (details are found in Sects."Clinical Applications of Stem Cell-Based Immunotherapy for Cancer"-"Advances in Overcoming Current Challenges").

**Table 1:** Summary of the applications, advantages, advances, and challenges of different types of stem cells

Feature	Hematopoietic Stem Cells (HSCs)	Mesenchymal Stem Cells (MSCs)	Induced Pluripotent Stem Cells (iPSCs)
Primary Application in Cancer Immunotherapy	Source for immune system reconstitution following radiation or chemotherapy. A starting point for modified immune cells such as NK cells and CAR-T cells	Inhibiting undesirable immune responses, delivering therapeutic medicines directly to tumors, and altering the TME to make it less conducive to malignancy	Producing a variety of immune cells tailored to each patient (such as CAR-T cells, NK cells, or tumor-targeting MSCs) for individualized treatments A source of"off-the-shelf"cell products
Key Advantages and Advances	Demonstrated clinical success in treating blood malignancies with bone marrow transplantation Vital for reestablishing immunological and blood systems following intensive therapies Can be modified to produce immune cells that fight cancer (e.g., CAR-T, CAR-NK)	Excellent drug carriers as they can naturally home to tumor sites Possess potent immune-modulating qualities that can aid in regulating immunological responses and inflammation Can be produced in high quantities and are simple to obtain from donors or from a patient's own body	Can be produced using a patient's own adult cells, eliminating immunological rejection and ethical issues with embryonic stem cells Possess the capacity to differentiate into a wide variety of cell types, providing therapeutic versatility. can be produced in high quantities
Major Limitations/Challenges	Significant risk of GVHD in allogeneic transplants Difficulty in finding perfectly matched donors for allogeneic use Complex and costly to expand and genetically modify outside the body for advanced therapies. Potential for long-term complications post-transplant	Their exact role in cancer is debated as they can sometimes help tumors grow instead of shrinking them, depending on the cancer type and environment It's difficult to make sure they consistently go only to tumor cells and stay there Difficult to fully control their immune modulating actions once inside the body to ensure consistent anti-cancer effects	There's a persistent concern about them forming tumors if any cells remain unspecialized after treatment The process of making iPSCs can sometimes cause unwanted changes in their genes Creating them is very expensive and involves complex manufacturing steps

**Fig. 1:**
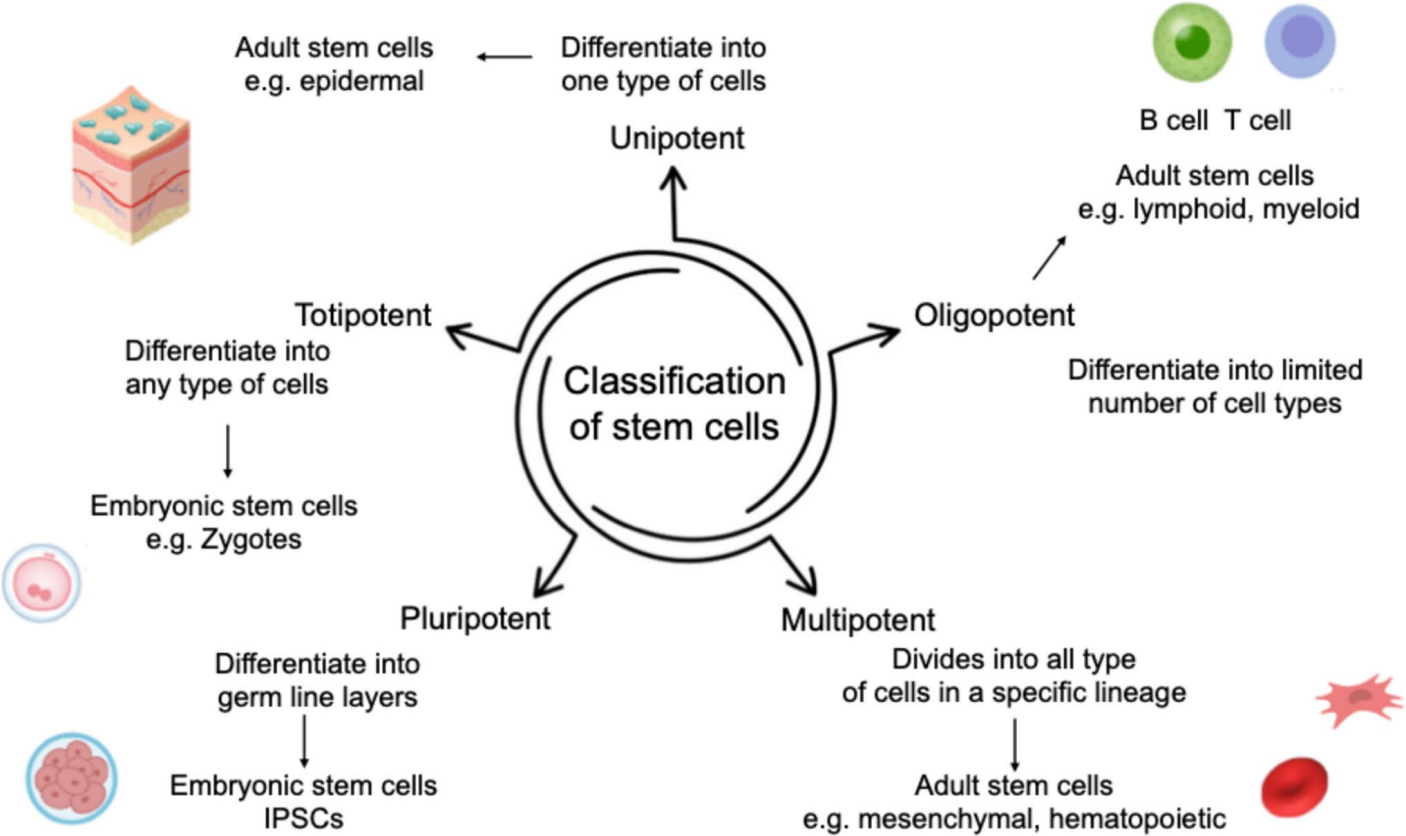
Classification of stem cells based on their differentiation (Created with BioRender)

Embryonic stem cells are capable of differentiating into any of the cell types found in an organism. They also undergo significant intrinsic replication stress and proliferate quickly [22]. Adult stem cells, found in diverse regions in the body, are undifferentiated cells that possess the capacity to proliferate, grow, and differentiate in order to replace cells that have undergone death or damage within a given tissue [23]. Adult pluripotent stem cells are reprogrammed embryonic stem cells created in laboratories. They have characteristics resembling those of embryonic stem cells, including the capacity for self-renewal and differentiation into several cell types [24]. Figure 1 shows the classification of stem cells based on their differentiation.

### Hematopoietic Stem Cells (HSCs)

**Fig. 2:**
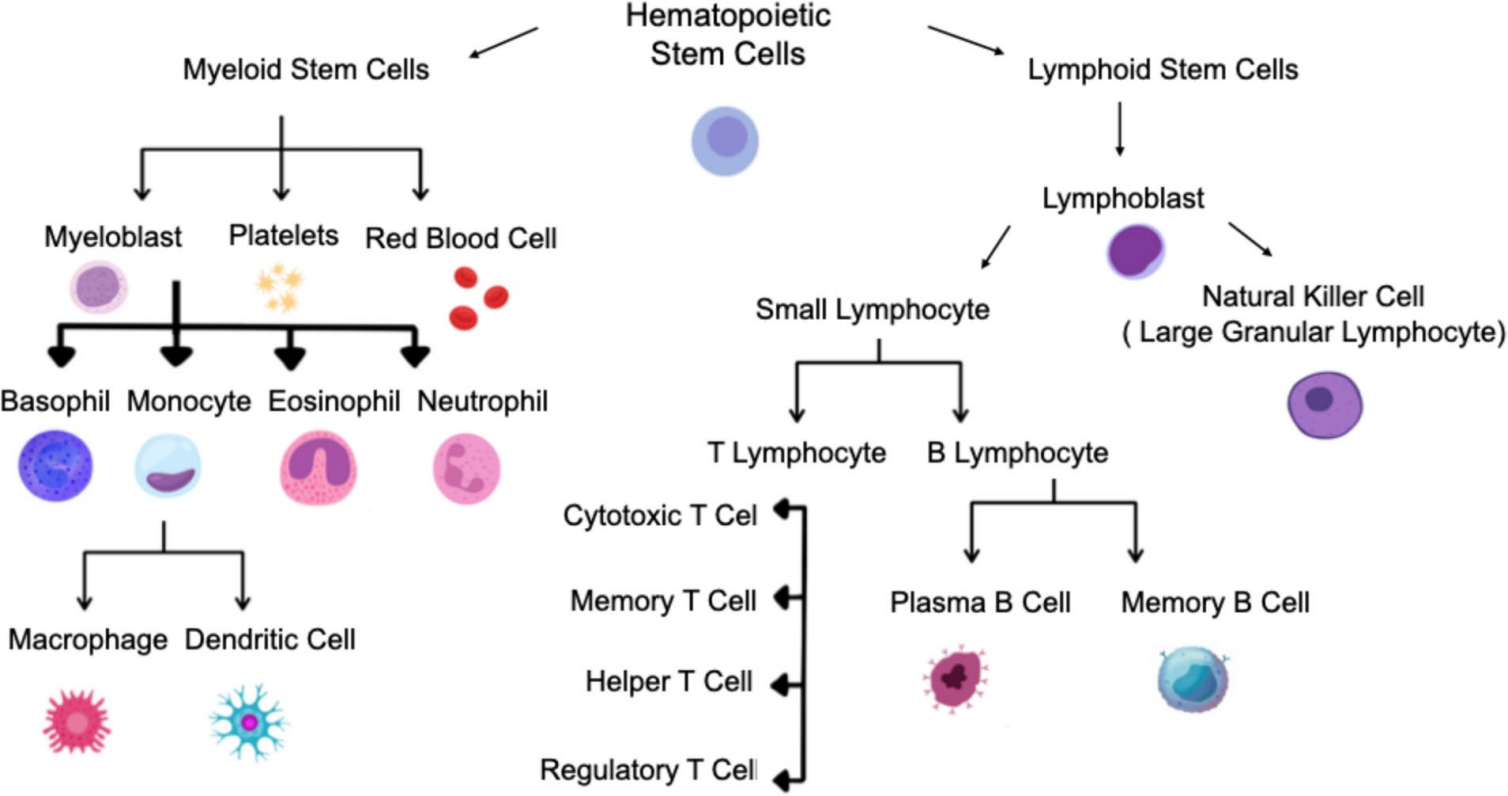
Cells produced from hematopoietic stem cells (Created with BioRender)

HSCs, located in the bone marrow, are a type of stem cells that have the capability to produce all the blood cells, including immune cells [25]. HSCs can differentiate into megakaryocytes, erythroid, and myeloid, including neutrophils and monocytes, or lymphoid cells, including B cells and T cells [26]. Using HSCs therapy is beneficial because it doesn't require cell expansion in culture or reconstitution of multicellular tissue architecture before transplantation [27]. The process of hematopoiesis sustains the ability of HSCs to renew themselves and the capacity to transform into different cell types. HSCs are used in various clinical settings, including replacing damaged bone marrow following aggressive chemotherapy or radiation [28]. D. T. Chu et al*.* (2020) state that blood-forming cells and leukocytes have been restored with HSC transplantation and utilized following intense chemotherapy or radiation treatment [20]. Figure 2 shows some types of cells that are produced from HSCs, including NK and T cells. Significant obstacles persist despite fundamental significance of HSCs in cancer therapy (more details found in Sect."Safety and Tumorigenicity Risks"). F. Hosseini et al*.* (2024) state that NK cells, originating from cord blood HSCs, have substantial anti-tumor activity and express a resilient cytotoxicity against cancer cells, rendering them a promising avenue for cancer immunotherapy [29]. Wahlster, L., & Daley, G. Q. (2016) discuss using human pluripotent stem cells to generate HSCs for treating various diseases through different strategies, including isolation from teratomas in vivo, directed differentiation, and direct transformation. They also discuss the improvement made in the production of various mature blood cells, such as red blood cells, white blood cells and platelets, from HSCs [30]. This accomplishment is essential to developing novel treatments for diverse diseases and disorders.

### Mesenchymal Stem Cells

MSCs represent a diverse group of rapidly proliferating cells that possess the capacity to differentiate into an array of non-hematopoietic cell types and can be extracted from both adult and fetal tissues. MSCs exhibit considerable promise in cancer immunotherapy due to their immunomodulatory features and capability to migrate to tumor locations. They may have a synergistic effect against tumors when they are combined with immunotherapy drugs, significantly improving cancer treatment [31]. Their primary function is to home tumors, regulate inflammatory and immune responses, regulate the efficacy of immunotherapy, and transmit immunomodulatory proteins [31]. MSCs are significant in delivering anti-cancer agents to tumor sites, inhibiting tumor growth, and adjusting the immunological environment around tumors. However, despite their tumor-suppressing capabilities, MSCs can promote tumor growth and metastasis by facilitating angiogenesis or inhibiting anti-tumor immune responses.

The unique capability of MSCs to travel to tumor locations and cooperate with the surrounding supportive microenvironment is a distinctive quality that led to the development of MSC-based experimental cancer cytotherapy, a procedure of hosting new cells in a tissue to treat diseases [32]. However, in some cases, MSCs can suppress tumors depending on their interaction within the TME [33]. Their specific role within the TME is explored in detail in Sect."Tumor Microenvironment Modulation". MSCs'natural tendency to home in on tumors can be harnessed to target and attack cancer cells. MSCs inhibit apoptosis and promote the persistence of different tissue cells through various pathways, including endogenous pathways, exogenous pathways, endoplasmic reticulum pathways, upstream regulatory pathways, pro-apoptotic pathways, and anti-apoptotic pathways [34]. Concerns also persist regarding their potential to differentiate into cells that could inadvertently support tumor progression, demanding rigorous quality control and monitoring in clinical applications.

### Induced Pluripotent Stem Cells

IPSCs provide a promising approach to cancer immunotherapy because of their capacity to differentiate into numerous types of cells involving immune cells, and their ability to be patient-specific, which helps minimize immune rejection concerns. IPSCs have the potential to generate customized tumor-targeting immune cells that can be precisely engineered to attack cancer cells. Because of their ability to be genetically engineered and self-renew, iPSCs offer a large-scale production for cell therapy [35]. They can be obtained from the somatic cells of the patient, such as blood cells and fibroblasts, and therefore possess the identical genetic and histological structure of the patient’s cells [36]. Utilizing these types of cells can create personalized immune cells with anti-tumor capabilities, which could be helpful in the personal treatment of cancer. Several organizations are currently working on producing immune cells from iPSCs for application in cancer immunotherapy [37]. However, the clinical application of IPSCS have major challenges that require thorough examination. A major concern is their genomic stability, as the reprogramming procedure can lead to genetic abnormalities, which raises concerns regarding long-term safety.

## Mechanisms of Action in Stem Cell-Based Immunotherapy for Cancer

**Fig. 3:**
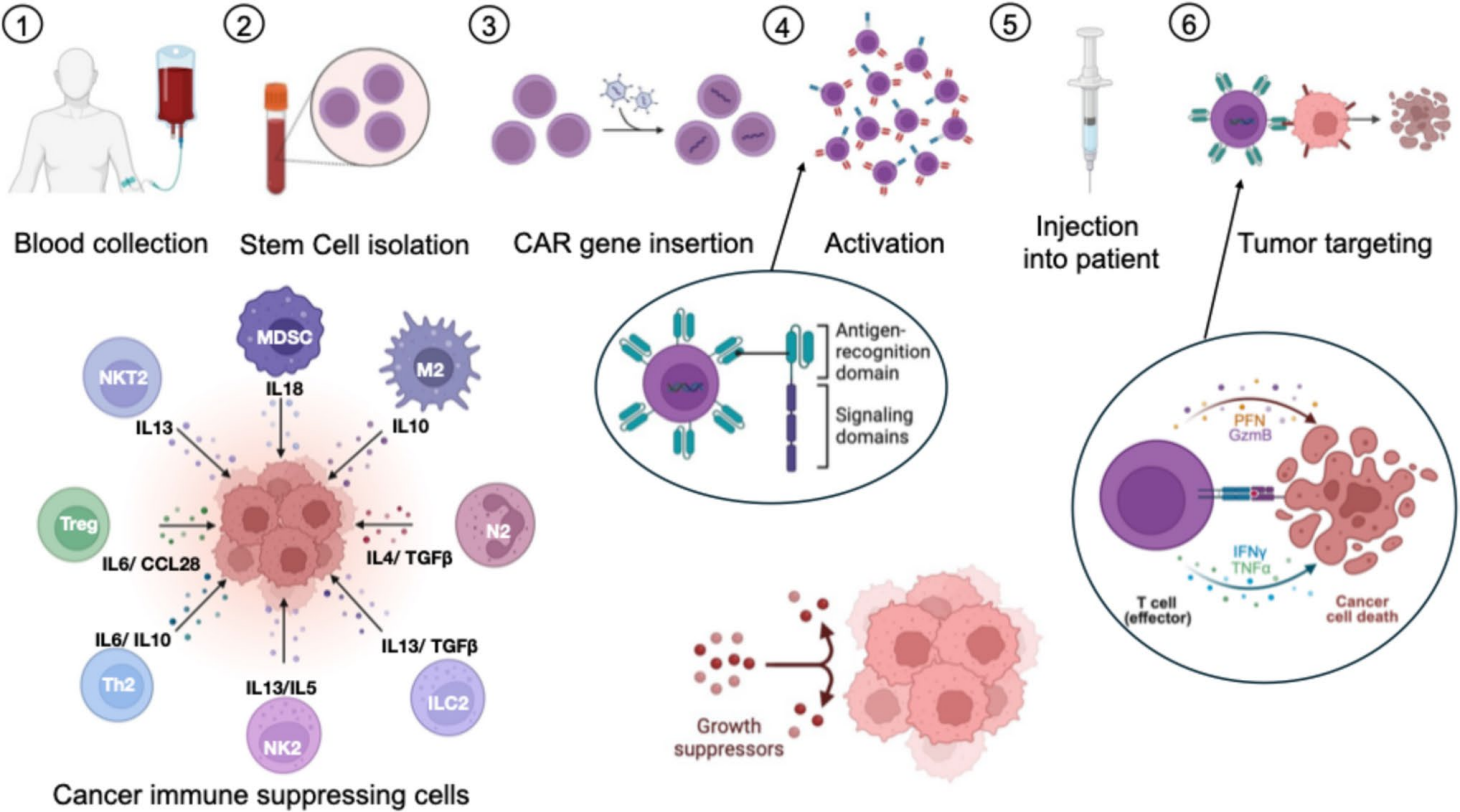
A summary of stem cell-based immunotherapy for cancer treatment. (1) Blood collection: blood is drawn from the patient. (2) Stem cell isolation: cells, including T cells, are isolated from the collected blood. (3) CAR gene insertion: T cells are genetically engineered to express a Chimeric Antigen Receptor (CAR) targeting specific tumor antigens. The CAR typically consists of an antigen-recognition domain (e.g., scFv) and one or more signaling domains for T cell activation. (4) Activation: The CAR T cells are expanded ex vivo to obtain sufficient numbers for therapeutic use. (5) Injection into patient: Activated CAR T cells are infused back into the patient. (6) Tumor targeting: CAR T cells recognize and bind to tumor cells, leading to their elimination. The effector mechanism involves the release of cytotoxic molecules such as perforin (PFN) and granzyme B (GzmB), and pro-inflammatory cytokines like interferon gamma (IFN-γ) and tumor necrosis factor alpha (TNF-α), ultimately inducing cancer cell death. The lower panel illustrates various cancer immune suppressing cells involved in TME: Myeloid-Derived Suppressor Cells (MDSC), M2 macrophages (M2), Regulatory T cells (Treg), T helper 2 cells (TH2), NKT2 cells (NKT2), N2 neutrophils (N2), Group 2 innate lymphoid cells (ILC2), and Natural Killer cells (NK2). These cells secrete various cytokines and chemokines, including interleukin (IL)−6, IL-10, IL-13, IL-5, transforming growth factor beta (TGF-β), and CCL2, which contribute to immune suppression. Growth suppressors are also depicted, indicating their role in inhibiting cell proliferation (Created with BioRender)

A visual summary of stem cell-based immunotherapy for cancer treatments are illustrated in Fig. 3 which are described in detail in in following sections.

### Tumor Microenvironment Modulation

**Fig. 4:**
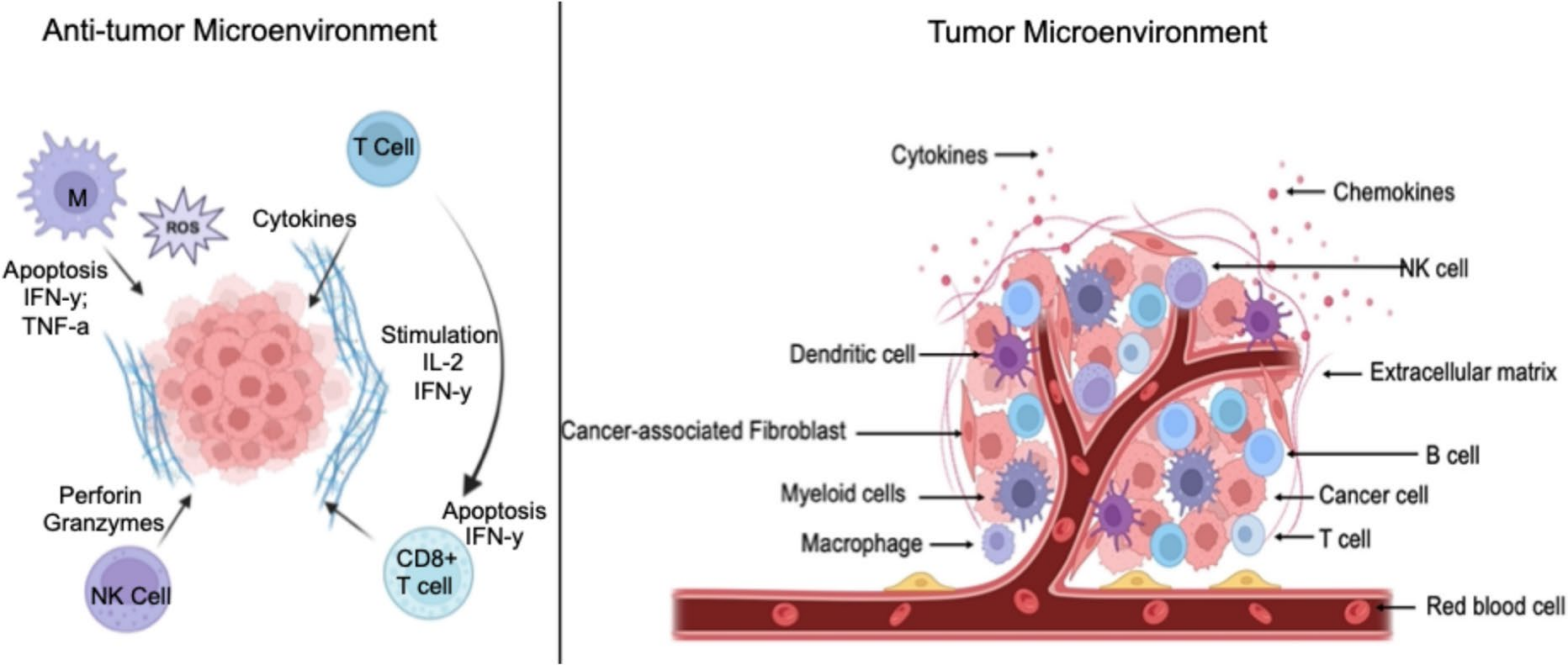
Illustration of the tumor microenvironment (TME) along with anti-tumor microenvironment. In the left panel, activated immune cells, such as T cells, CD8 + T cells, and Natural Killer (NK) cells, interact with cancer cells. These cells induce apoptosis in cancer cells through mechanisms involving the release of Perforin and Granzymes, and the production of Reactive Oxygen Species (ROS). Cytokines such as Interleukin-2 (IL-2), Interferon gamma (IFN-γ), and Tumor Necrosis Factor alpha (TNF-α) are involved in stimulating and mediating anti-tumor responses. In the right panel, a comprehensive representation of the cellular and structural components of the TME (Created with BioRender)

TME plays a critical role in shaping the success or failure of cancer immunotherapy. It profoundly influences the characteristics of the Cancer stem cells (CSCs) as they work in this environment by secreting growth factors, inflammatory factors, and microRNAs [38] CSCs are a small group of malignant cells that can renew themselves and give rise to new types of malignant cells. These cells are important in tumor initiation, as are their metastasis ability and resistance to classical therapy [38]. A TME not only contains a group of cancer cells but is also composed of cellular and noncellular components such as signaling pathways and cytokines [38]. Furthermore, it contains immune cells, stromal cells, blood vessels, molecules, and extracellular matrix that control tumor progression and development [39]. Figure 4 represents the complexity of the TME, including signaling molecules and different types of cells, such as immune cells, stromal cells, and endothelial cells. It shows cancer cells surrounded by various types of cells, such as fibroblasts, red blood cells, macrophages, NK cells, and CAR-T cells. It also shows the extracellular matrix and molecules like cytokines and chemokines surrounding the tumor.

With their distinctive immunological properties, cells in the TME will have the ability to determine if the tumor will survive and affect the neighboring cells [40]. Several factors in the TME could promote CSC properties, including VEGF and TGF-β factors, an extracellular matrix, immune modulation, and providing a niche to CSCs [38]. The TME greatly influences the behavior of cancer cells, including their growth, spread, and resistance to treatment. Furthermore, it can promote the development of blood vessels, which provide oxygen and nutrients to the tumor, and suppress the immune system response, allowing the tumor to grow unhindered [39].

TME affects the development and growth of cancer, including different interacting cell types like immune cells and MSCs through the extracellular matrix and soluble factors in addition to the corresponding tumor cells [41]. Both in vivo and in vitro trials have shown inflammation regulation when MSCs interact with innate and adaptive immune cells [42]. In vitro, MSCs polarize macrophages to an anti-inflammatory M2 phenotype and suppress T-cell proliferation [43, 44]. In contrast, the outcomes of in vivo studies show that MSCs did not increase survival in pancreatic cancer, presumably because of context-dependent TGF-β secretion, even though they decreased GVHD in Phase II trials [45]. Moreover, pre-clinical and clinical findings have displayed that MSCs can modulate the TME by suppressing cancer cells and determining the final site of tumor cells [46]. MSCs can suppress tumor angiogenesis, which is blood vessels that help in the development and spread of tumors [46]. MSCs can either promote or inhibit tumor progression through various mechanisms by secreting soluble factors that can modulate the immune system [43, 47]. MSCs inhibit the polarization of macrophages to M1 while favoring their polarization to M2. They can also inhibit the activation, differentiation, and effector functions of NK cells and antigen-presenting cells (DCs) using prostaglandin E2 (PGE2) protein [43]. In the presence of interleukin 6 (IL-6) and granulocyte-monocyte colony-stimulating factor (GM-CSF), MSCs can influence macrophage function while transforming growth factor beta (TGF-β) and indoleamine 2,3-dioxygenase (IDO) inhibit NK cell function, leading to immune enhancement [43]. MSCs have immunomodulatory properties and are able to modify the TME to promote immune cell infiltration, reduce immune suppression, and enhance anti-tumor immunity [44]. Their immunomodulatory mechanism can either be through direct contact with immune cells or by secreting different molecules such as cytokines, chemokines, and growth factors [48].

Moreover, MSCs possess the ability to modify and change the behavior of cells within the TME, including the induction of an anti-inflammatory effect, inhibition of tumor angiogenesis, and the prevention of metastasis [49]. The ability of MSCs to load and release chemotherapeutics at the site of tumors is a preferred option for targeted drug delivery as it reduces the unexpected side effects of chemotherapeutics and improves clinical outcomes [49]. As a result, to control malignancies and achieve more favorable health outcomes during cancer treatment, cancer cells need to be targeted and manipulated [40]. The strategic alteration of the TME by MSCs offers a compelling pathway for improving cancer immunotherapy; however, the inherent complexities of the environment present significant obstacles that require more investigation. The TME often serves as a critical physical and immunological barrier, defined by dense stromal elements, severe hypoxia, and a high presence of immunosuppressive cells such as regulatory T cells and myeloid-derived suppressor cells. Current strategies frequently face limitations in consistently achieving widespread penetration, sustained therapeutic agent delivery, or complete reversal of the TME's immunosuppressive state across diverse tumor types, highlighting the need for more sophisticated and durable intervention approaches.

### Stem Cell-Derived CAR-T and NK Cells

**Fig. 5:**
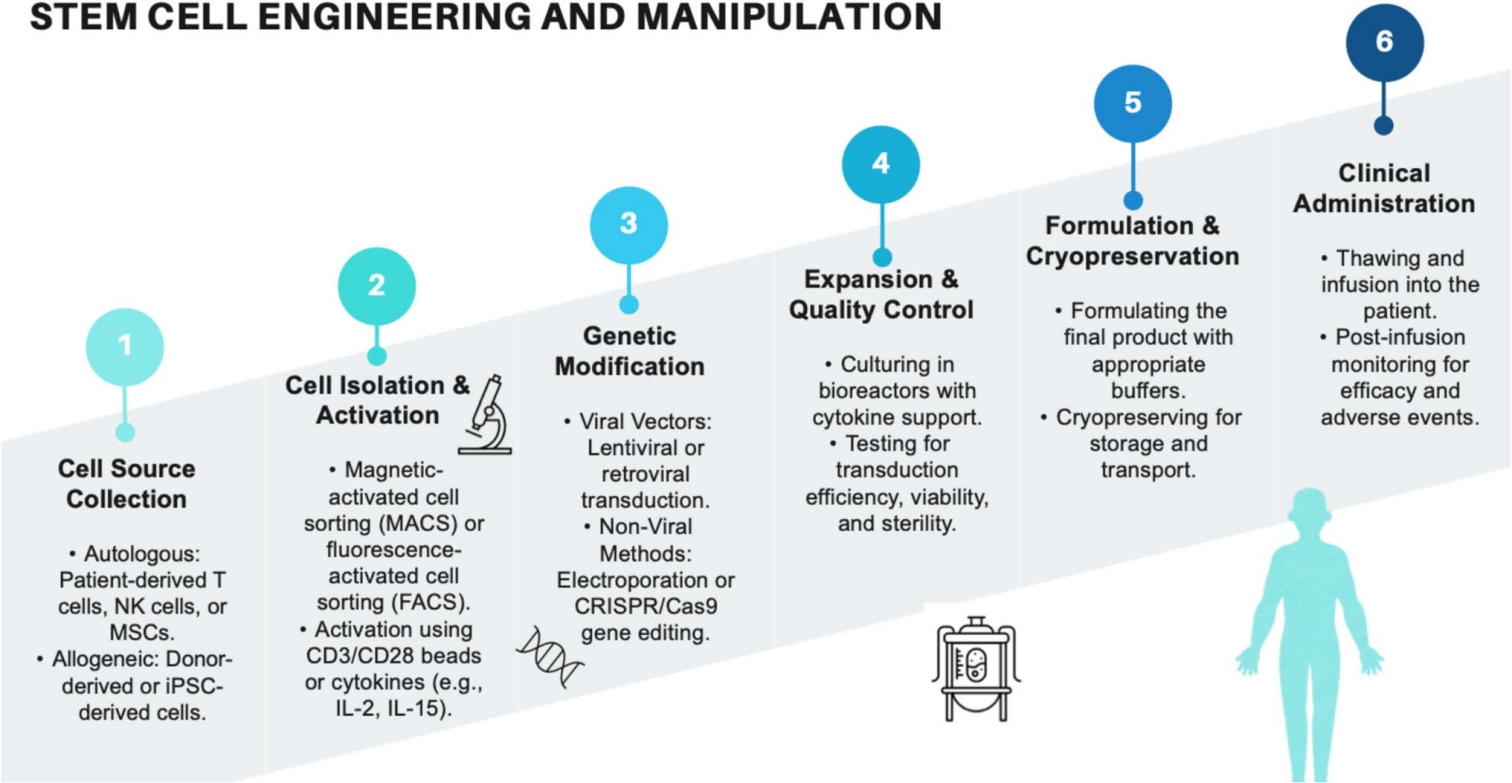
Core stages in stem cell engineering and manipulation

Referring to Fig. 5**,** it presents the core stem cell engineering phases and manipulation in general. Current developments aim to reduce the manufacturing period from weeks to days, enhancing patient accessibility. iPSCs are used in cancer immunotherapy because of their ability to develop immune cells like CAR-T and NK cells that can be engineered to target specific cancer antigens. CAR-T cell treatment is a type of immunotherapy that includes the modification of T cells genetically to generate artificial chimeric receptors that contain T cell-activating regions and antigen-binding regions [50, 51]. T cells can target cancer cells by recognizing foreign proteins on their surface, called cancer antigens, and designing special receptors that recognize them [51]. The two principal approaches to T cell immunotherapy are HLA-restricted and HLA-unrestricted. HLA-restricted immunotherapy employs natural TCRs that recognize cancer antigens bound to HLA molecules. In contrast, HLA-unrestricted immunotherapy utilizes CAR-Ts that recognize cancer antigens directly, negating the necessity for HLA molecules [51].

CAR-T cells can be engineered to produce and release CAR protein, which binds to a particular protein on the surface of the tumor; then, they are expanded and reintroduced to the patient. This technique enables CAR-T cells to identify and destroy cells demonstrating the specific antigen effectively [52]. CARs have four domains with particular functions: an antigen-binding domain, a hinge region, a transmembrane domain, and an intracellular domain. The intracellular signalling domain triggers the T cells after antigen engagement, the extracellular antigen-targeting moiety such as scFv, confers specificity, and the transmembrane and hinge domains secure the receptors to the cell surface and project the scFv into the extracellular area [53]. Modifying the structures of the domains allows for different functionalities of the CAR [54]. While CAR-T cells have shown early success against certain types of blood cancers, major barriers such as toxicity and tumor relapses in antigen-negative tumors currently limit the broader use of CAR-T cells as a cancer therapy. These challenges are amplified in solid tumors, where distinct tumor antigens may not be available, and the immunosuppressive nature of the TME is a potential barrier to CAR-T cell function. T cells can also be reprogrammed through gene transfer with genetic modules that enhance their therapeutic potency and specificity to eliminate these challenges [53]. Bioluminescence imaging was used to evaluate tumor suppression in preclinical experiments, and RECIST criteria were used in clinical trials. Preclinical studies demonstrate that CAR-NK cells eliminate CD19 + hematologic tumors in murine models. According to the study, 14 days after injection, the tumor volume has decreased by 75% [55]. However, clinical efficacy is restricted to early-phase trials for B-cell malignancies, with minimal activity in solid tumors due to inadequate infiltration [56].

**Fig. 6:**
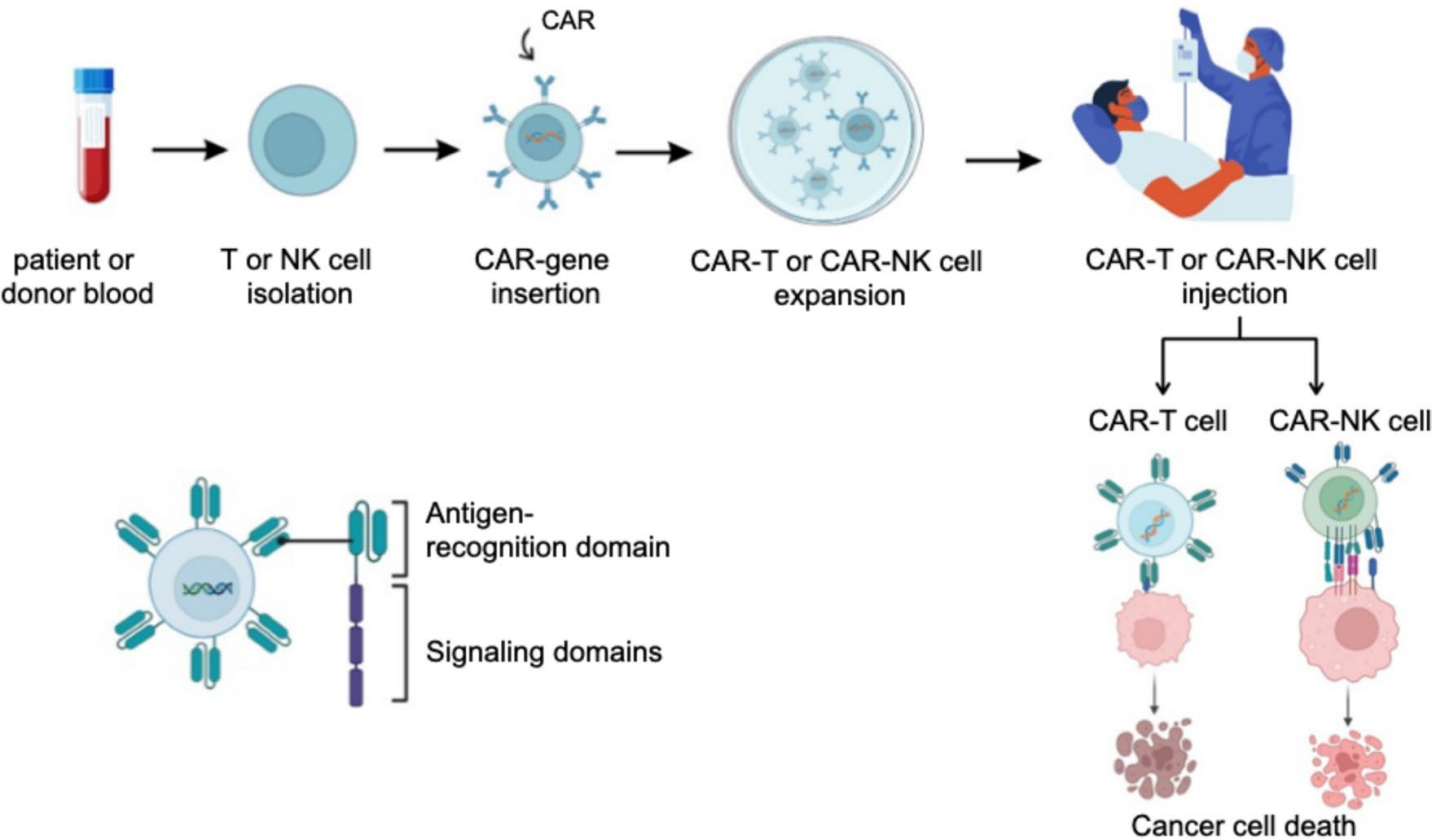
Treatment using chimeric antigen receptor T cells (CAR-T) or natural killer cells (CAR-NK). The treatment starts with isolating T or NK cells from the patient's or donor's blood. Then, cells are genetically modified to express CARs. After that, CAR-T or CAR-NK cells are expanded to a desired number. Finally, CAR-T or CAR-NK cells are injected into the patient’s body to kill cancer cells (Created with BioRender)

On the other hand, NK cells are a type of white blood cell that plays a vital and essential role in the immune system as they can attack and destroy cancer cells [57]. They can be created from iPSCs, which are stem cell sources that can repair themselves limitlessly and can be modified genetically to enhance NK cells’ killing specificity, potency, and effectiveness in vivo and later in the clinic [55]. NK cells lack antigen-specific receptors and are powerful innate immune system effector lymphocytes with several mechanisms to kill cancer cells [57]. They play a significant role in tumor surveillance, making them the next-generation tool for adoptive immunotherapy [57]. These cells are specialized immune effectors essential in tumor immunosurveillance due to their distinctive capacity to identify and eliminate abnormal cells using a complex array of germline-encoded receptors without the need for prior sensitization [58]. NK cells become activated when their receptors interact with the target cells, releasing cytotoxic granules that lyse tumor cells directly (Fig. 6) [59]. Utilizing NK cells in treatment includes separating NK cells from either the patient's blood or from a giver, adjusting them genetically, and infusing them back into the patient's body to fight cancerous cells [58]. Another treatment includes utilizing NK cells directly from the patient's blood without any genetic alteration. NK cells can also be combined with other therapies or drugs, such as immunotherapy or chemotherapy, to upgrade the impact of the treatment [58]. Various strategies have been investigated to enhance the efficacy of NK cells in cancer therapy. These include using cytokines and synthetic compounds to augment their proliferation and killing capacity, targeting immune checkpoints, incorporating CARs to enhance cancer specificity, and genetically removing inhibitory molecules [57]. CAR-NK cells can treat different types of tumors, including hematological and solid tumors, because of their advantages, such as safety, speed, broad targeting, and multi-functionality. Although most studies are still in the pre-clinical stage, some early clinical trials have yielded promising results [56].

### Oncolytic Virotherapy

Oncolytic virotherapy is used to utilize the virus to target and destroy cancer cells [60]. Recently, oncolytic virotherapy has exposed considerable potential in cancer therapy, as the oncolytic virus can eliminate cancer cells without harming healthy tissues via virus self-replication and anti-tumor immune response [61]. Oncolytic virus therapy can be combined with other treatments, such as CAR-T cell therapy, to enhance the effectiveness of the treatment and expand its scope of use. For instance, oncolytic viruses can improve the ability of CAR-T cells to reach and destroy tumors [61]. Oncolytic viruses are viruses that multiply or replicate selectively and kill cancer cells without causing damage to healthy tissues, whether they arise naturally or are created through genetic engineering [62, 63]. A comprehensive overview of their types, mechanisms, clinical applications, engineering strategies, advantages, current challenges and emerging research trends is provided in Table 2. The first and most potent virus used in oncolytic virotherapy is human adenovirus. However, ongoing research has suggested that other viruses, such as herpes simplex and measles, could also be considered potential candidates in cancer therapy [64]. The oncolytic virus based on the herpes simplex virus, T-VEC, has completed a phase III clinical trial and has been approved by the US Food and Drug Administration (FDA) for use in cancer biotherapy [64]. Furthermore, the vaccine strain of the measles virus has shown impressive results in pre-clinical and clinical trials [64]. Due to their capability to identify and target tumors, stem cells can facilitate the delivery of oncolytic viruses, which are specifically engineered to infect and kill cancer cells while simultaneously stimulating an anti-tumor immune response. TMEs express pro-tumorigenic molecules that promote tumor growth. They can produce chemoattractant gradients surrounding the TME, leading stem cells to move to the tumor locations [65].

**Table 2:** Summary of the key details of oncolytic viruses

Aspect	Details	References
Definition	Oncolytic viruses are engineered or naturally occurring viruses that selectively infect and lyse cancer cells without harming normal tissues	[63]
Types of oncolytic viruses	- **Herpes Simplex Virus (HSV):** High replication rate, can deliver transgenes (e.g., T-VEC often targets solid tumors), and highly modifiable - **Adenovirus:** Effective in solid tumors, used in clinical trials, well-characterized, non-integrating, often used for gene therapy and systemic delivery (e.g., ONCORINE H101) - **Reovirus:** Exploits Ras-signaling defects in cancer cells, and less immunogenic - **Measles Virus:** Highly immunogenic, attenuated vaccine strains, shows promise in myeloma and ovarian cancer, and capable of systemic delivery to tumors	[62, 63]
Key features	- **Tumor Selectivity:** Targeting cancer-specific signaling pathways (e.g., defective interferon response) - **Immune Modulation:** Stimulates anti-tumor immune responses - **Genetic Modifications:** Increase safety and specificity	[62]
Anti-tumor Mechanisms	- **Direct Lysis:** Viral replication destroys cancer cells - **Immunostimulant:** Releases tumor-associated antigens to activate immune responses - **Angiogenesis Inhibition:** Disrupts tumor vasculature	[62, 63]
Clinical applications	- Approved therapies include **T-VEC** (HSV-based) for melanoma - T-VEC show promising results in glioblastoma, with specific candidates like oHSV G47Δ (approved in Japan for recurrent glioma), and ongoing trials with other oHSVs, adenoviruses, and polioviruses - Promising results in lung cancer (with ongoing trials such as MEM-288 (adenovirus-based) for late-stage NSCLC.), and hepatocellular carcinoma (e.g., VG161 in promising Phase I/II trials, and H101 approved in China) - Often used in combination therapies with immune checkpoint inhibitors	[60, 63]
Engineering Strategies	- Insertion of immune-stimulatory genes (e.g., GM-CSF) - Removal of virulence genes to enhance safety - Retargeting viral tropism to specific tumor markers (e.g., integrins, HER2)	[66]
Advantages	- **Specificity:** Targets cancer cells with minimal effects on healthy tissues - **Dual Action:** Direct lysis and immune activation - **Adaptability:** Can be modified for personalized therapies	[62, 63]
Challenges	- **Immune System Neutralization:** Host immunity may clear the virus - **Heterogeneity:** Tumors vary in susceptibility - **Delivery Limitations:** Difficulty in reaching metastatic or dense tumors	[61, 63]
Emerging Research	- Exploring non-human viruses like Newcastle disease virus, and exploring new viral backbones (e.g., vaccinia virus, Seneca virus) for broader tropism and improved safety - Combining OVs with nanotechnology for enhanced delivery (synergistic effects with immune checkpoint inhibitors, chemotherapy, and radiation to enhance anti-tumor responses) - CRISPR-Cas systems for precise genetic engineering of viruses, targeted tumor specificity, enhanced replication, and arming viruses with therapeutic genes. (e.g., tumor-specific promoters)	[62]

MSCs, neural stem cells (NSCs), and adipose-derived stem cells have been investigated as potential cell transporters for oncolytic virotherapy, and they led to different results as each kind of stem cell has its strengths and weaknesses [65]. Studies have shown that combining oncolytic viruses with MSCs increases the effectiveness of treatment and reduces side effects because of the ability of MSCs to reach tumors quickly [66]. Experiments on mice have shown promising results in treating brain tumors and metastatic cancers [66]. While some oncolytic viruses have been approved to treat certain types of cancer, such as melanoma, several oncolytic viruses are currently being tested in clinical trials to treat other types of cancer. MSC-loaded oncolytic adenoviruses produced 60% tumor shrinkage in glioblastoma xenografts [66]. On the other hand, 70% of patients in a Phase I trial experienced viral clearance by neutralizing antibodies, which limited tumor delivery. There was no response in colorectal metastases, and the effectiveness varied depending on the type of tumor [67].

New generations of oncolytic viruses are also being developed that more precisely target cancer cells, with fewer side effects [67]. Oncolytic viruses are a promising tool in cancer treatment, as they can be used alone or in combination with other treatments such as chemotherapy or immunotherapy. However, challenges remain with this type of therapy. As discussed in the challenges section of Table 2. One major issue is the immune system itself, which can quickly attack and eliminate the therapeutic viruses before they get to the tumor, reducing their efficacy. Delivering these viruses efficiently throughout the body is another significant hurdle. Even though these viruses are designed to target cancer cells, there are still concerns about unintended effects or the virus spreading to healthy tissues, highlighting the need for improved safety measures. Additionally, tumors are highly diverse, meaning some cancer cells may resist viral infection, leading to inconsistent treatment responses [61, 63]. To overcome these obstacles, researchers are working on advanced virus engineering, better delivery methods, and combination therapies that enhance precision while reducing immune-related side effects [62, 66]. These strategies are comprehensively summarized in Table 2**,** which presents the ongoing development of oncolytic virotherapy includes advanced genetic engineering to improve selectivity and decrease off-target effects, as well as investigating its potential for synergy in combination therapies with other immunotherapeutic drugs. The goal is to make this innovative treatment safer and more effective for patients [61].

## Clinical Applications of Stem Cell-Based Immunotherapy for Cancer

**Fig. 7:**
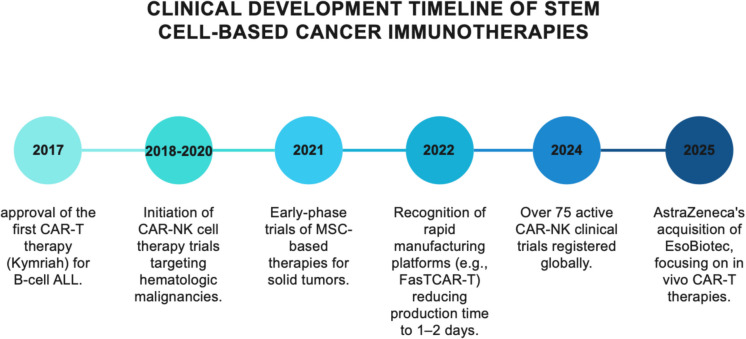
Clinical development timeline of cell-based cancer immunotherapies

Clinical applications of stem cell-based immunotherapy for cancer have developed rapidly in the past few years. Figure 7 shows a timeline that illustrates the rapid evolution of cell-based immunotherapies in oncology.

### CAR-T Cell Therapy Enhancement for Blood Cancers

A study conducted by Freiwan et al*.* (2022) demonstrates that naturally existing CD7-negative T cells present a viable substitute for CAR-T cell treatment and provide the possibility for effective cancer cell targeting while simultaneously lowering the risk of side effects [68]. The same study shows that these cells effectively kill cancer cells in laboratory settings when engineered with a CAR that targets cancer cells. The treatment of leukemia and lymphoma with CAR-T cells represents a highly promising use of stem cell-based immunotherapy, as it has shown improved overall survival compared to traditional treatments. CAR-T therapy has occasionally been observed to induce total remissions, with no evidence of the cancer returning [69]. A study examining trends in leukemia incidence at global, regional, and national levels between 1990 and 2017 suggests that although leukemia incidence has declined globally, incidence rates of certain types of leukemia, such as acute myeloid leukemia (AML) and chronic lymphocytic leukemia (CLL), have increased significantly in most countries. The number of new cases increased from 354.5 thousand in 1990 to 518.5 thousand in 2017; however, the age-standardized incidence rate decreased by 0.43% per year in leukemia incidence [70]. Leukemia constitutes a variety of threatening disorders of blood and bone marrow with the probability to be life-threatening [70]. CAR-T cells must be within the body for an extended period to achieve persistent remission in leukemia, as these cells function as a form of immunotherapy, boosting the immune response, preventing relapse, and improving overall survival [71].

Axicabtagene ciloleucel therapy represents a targeted CAR-T cell therapy for patients with aggressive B-cell lymphomas who have not responded well to conventional treatments. The therapy involves the collection of a patient's T cells, which are then genetically modified to express CARs, which enable the cells to recognize and attack the CD19 protein on lymphoma cells, with the goal of eliminating cancer [72]. The use of CAR-T cells therapy has demonstrated encouraging results in the treatment of hematologic cancers; nevertheless, additional research is necessary to enhance the effectiveness and safety of this therapy and extend its application to solid tumors [69]. Research shows that CAR-T cells targeting CCR4 receptors, found abundantly on the surface of T-cell lymphoma cells, are effective in attacking and destroying T-cell lymphoma cells. The same research indicates that treatment with CAR-T cells targeting CCR4 has limited side effects compared to conventional treatments [72]. Using CAR-T cells in treatment is considered a major choice for treating different types of cancers, particularly blood cancer; nevertheless, clinical trials have faced numerous issues, including disease recurrence and refractory cases [54].

Even with the success of CAR-T cell therapy, issues such as primary resistance remain significant concerns. Additionally, severe and often deadly toxicities require close observation and specialized care, which limits their use and accessibility outside of highly specialized hospitals. To develop safer and more broadly applicable CAR-T cell approaches, it is imperative to comprehend the mechanisms underlying these restrictions.

### Stem Cell-Mediated Drug Delivery for Solid Tumors

MSCs can deliver cancer drugs directly to cancer cells. They are highly efficient foundations for a cell-mediated drug delivery system in a variety of pre-clinical and clinical investigations [73]. The advantages of utilizing human MSCs as drug carriers include autonomous propagation, the capacity to differentiate into diverse cell types, and immunoregulatory properties. While the challenges associated with their use as drug carriers include issues related to loading efficiency, precise targeting, and toxicity [73]. MSCs are highly relevant to cell-based therapies used in cancer therapy because of their capacity to target tumors and modulate the immune response [74]. MSCs have a low level of autoimmunity due to the lack of co-stimulatory molecule expression, meaning that immunosuppression is not required during allogeneic cell transplantation [75]. The affinity of MSCs for damaged tissues and tumor sites makes them a promising vector for delivering therapeutic agents to tumors. MSCs can be genetically modified using viral vectors to encode tumor suppressor genes, immunomodulatory cytokines and their combinations, and other therapeutic approaches, including mobilizing MSCs with chemotherapeutics or nanoparticles [75]. A study was conducted to examine the effectiveness of this approach in delivering the TRAIL protein (tumor necrosis factor-related apoptosis-inducing ligand). This protein has the ability to induce cancer cell death in TRAIL-resistant breast cancer cells. The findings demonstrated that MSCs could effectively carry TRAIL to cancer cells and trigger cell death even in resistant cells [75]. This finding suggests that MSC-based delivery could be useful in overcoming resistance to cancer therapies. Another study concentrates on the use of MSCs as drug carriers to deliver the chemotherapy drug doxorubicin to lung cancer metastases. According to the study, MSCs loaded with doxorubicin were able to effectively target and shrink melanoma metastases in the lungs of mice, resulting in a significant reduction in tumor size and an increased survival time in the treated cohort as opposed to the control cohort [76].

Even though MSCs show promise as customized drug delivery vehicles, several major challenges remain to be addressed. One of the biggest challenges is loading therapeutic medicines into MSCs efficiently while ensuring that they regularly reach and stay at tumor sites. Controlling the method and timing of these drugs'release throughout the body presents technological difficulties. Moreover, Concerns regarding the long-term effects of MSCs on patients and potential toxicity from the drugs that are given must be addressed by researchers. Further study is necessary to overcome these obstacles and fully realize MSCs'potential for safe, effective treatments.

### Combining Stem Cells with Other Cancer Treatments

Since CAR-T cell therapy has some limitations, combining CAR T-cell therapy with other therapeutic approaches can help address and overcome these limitations and increase its efficacy. This can be achieved by combining CAR-T cell therapy immune checkpoint inhibitors (ICIs), bi-specific T-cell engagers (BiTEs), and antibody–drug complexes (ADCs) [77]. Combining chemotherapy with CAR-T cell therapy to treat solid tumors has presented a possibility to be an efficient treatment, as solid tumors are generally less responsive to CAR-T therapy than hematologic tumors or blood malignancies. Chemotherapy reduces immunosuppressive cells, induces an inflammatory TME, and weakens the tumor stroma, which may improve the ability of the tumor to support CAR-T cells [78].

The combination of radiation therapy (RT) with CAR T-cell therapy provides a potential option to improve the ability to control tumors and reduce treatment toxicity in the treatment of lymphoma [79]. Prior to CAR T-cell administration, radiation therapy can help in the reduction of the tumor burden, which will make it easier for targeted immune cells to identify and kill cancer cells. Furthermore, the strategic use of radiation therapy may help in the reduction of the potential side effects of CAR T-cell therapy [79]. Another study suggests that combining radiation therapy (RT) with CAR-T cell therapy, which involves using low-dose total body irradiation (LD-TBI) before giving CAR-T cells, can significantly enhance the treatment's efficacy in treating acute lymphoblastic leukemia (ALL) [80].

The combination of ICIs and MSCs for cancer treatment might result in a synergistic effect, potentially leading to improved outcomes compared to either therapy alone [81]. While ICIs permit the immune system to identify and fight cancer cells by blocking immune checkpoints, MSCs have the ability to produce factors that inhibit inflammation and promote tissue repair to reduce the side effects of ICI treatment [81] Table 3 summarizes some of the cancer treatments that combine stem cells to improve therapeutic outcomes and reduce side effects.

**Table 3:** Combination of stem cells with some cancer treatments to improve outcomes and reduce side effects

Combination	Mechanism	Current status	Reference
Stem cell with gene editing (CRISPR-Cas9) for blood cancer	Genetically modifying HSCs to enhance immune response using CRISPR-Cas9	Early-phase clinical trials	[82]
Stem cell with CAR-T therapy for blood cancer	Engineering CAR-T cells to improve immune attack with the support of stem cells	Approved by FDA for blood cancer. Ongoing studies for solid tumors	[83]
Stem cell with tumor-infiltrating lymphocytes (TIL) for solid tumors	Harvesting, growing, and reintroducing TIL cells and using stem cell therapy to boost immune response	Approved by FDA for melanoma. Ongoing studies for other solid tumors	[83]
Stem cell with radiation therapy for solid tumors	Using HSCs to reduce the damage that is caused by radiation, repair tissues, and improve immune targeting	Pre-clinical and early-phase trials	[82]

## Challenges in Stem Cell-Based Immunotherapy for Cancer

**Fig. 8:**
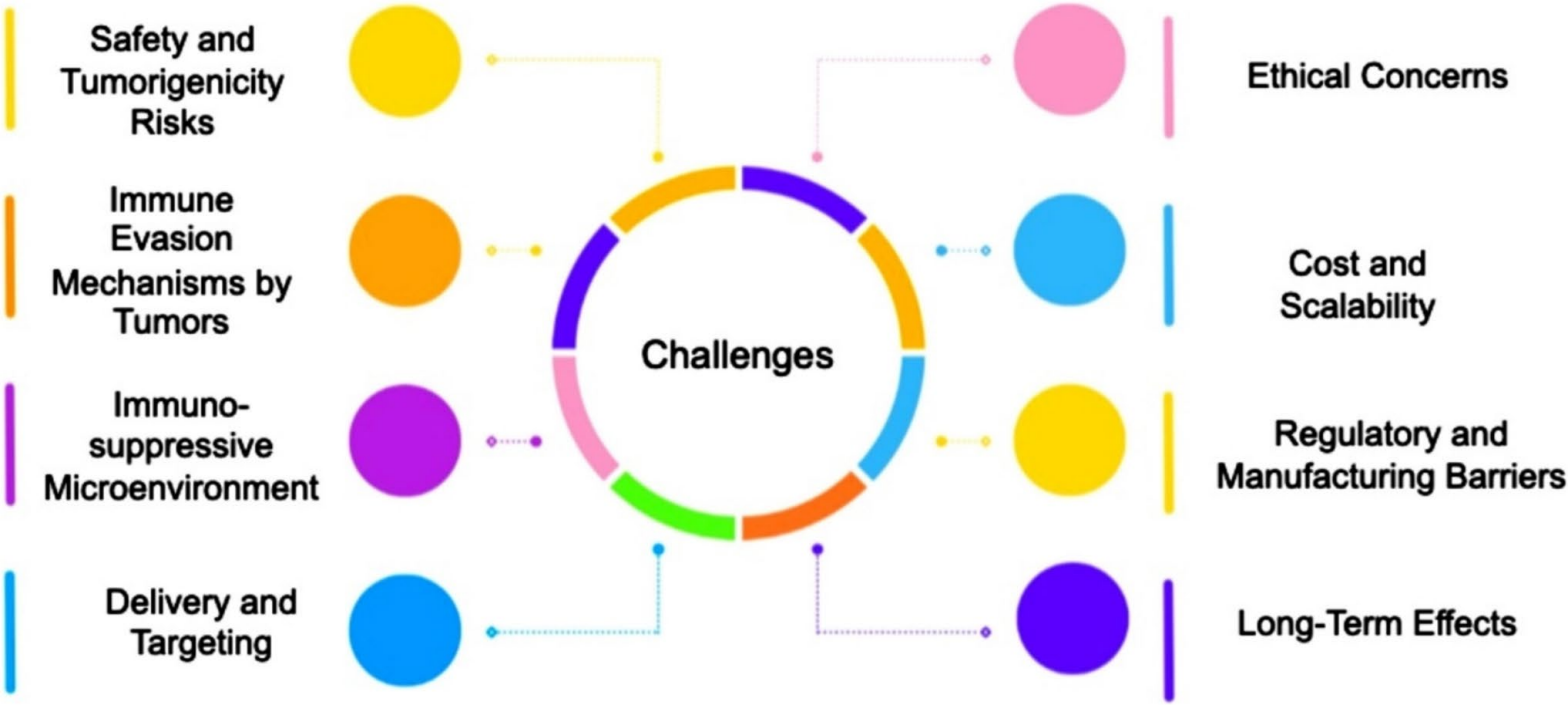
Challenges in stem cell-based immunotherapy for cancer (Created with BioRender)

The combination of stem cell therapies and immunotherapy represents a potentially effective approach to cancer treatment as it has the potential to improve the capacity of the immune system to attack cancer. Figure 8 summarizes some of the challenges in stem cell-based immunotherapy for cancer. It is perceived that various obstacles still need to be overcome before stem cell-based immunotherapy can be used to treat cancer on a large scale. These challenges include safety and tumorigenicity risks, tumour immune evasion mechanisms, and regulatory and manufacturing obstacles.

### Safety and Tumorigenicity Risks

Even though stem cell-based immunotherapies hold a great potential for the treatment of cancer, there are several significant obstacles related to safety and the potential for stem cells to become tumor cells. Thorough and careful pre-clinical research is necessary to evaluate the risks because of the potential of stem cells to develop into different cell types or possibly stimulate tumor growth. For example, several findings have revealed that MSCs are able to become tumor cells in some cases, which may contribute to the spread of tumors despite their ability to treat tumors [84]. This occurs due to specific changes in their genes or their environment. Another example is that allogeneic hematopoietic stem cell transplantation (HSCT) has substantial risks of GVHD, which can be severe and life-threatening, and is limited by the availability of perfectly matched donors. Additionally, MSCs have immunosuppressive properties that may make it further challenging for the immune system to fight cancer cells. Furthermore, the unfavorable surrounding conditions of the tumor in the microenvironment may disturb the capacity of MSCs to survive and perform their required functions [84].

Moreover, there are significant challenges in terms of scalability, cost, and ensuring long-term engraftment and functionality without adverse effects due to the complexity of the ex vivo expansion and genetic modification of HSCs for novel therapeutic applications, such as the production of engineered immune cells.

Researchers are working on improving the safety of these cells. This includes carefully cleansing the cells to ensure that only the proper type is employed, or even introducing genes into the cells that function as an internal switch if the cells start acting inappropriately. To ensure safety, extensive testing is conducted in the lab prior to any treatment being administered to humans. Other safety concerns include the rejection of allogeneic stem cells by the patients’ body because it perceives them as alien.

### Immune Evasion Mechanisms by Tumors

Tumor cells, including CSCs, represent a tiny proportion of tumor cells that possess the capacity for differentiation and self-renewal [85, 86]. Novel axes like CD155–CD96 are becoming more widely acknowledged for their roles in immune suppression. Recent findings demonstrate that deficiency in this pathway can regulate IL-9 production, emphasizing how checkpoint imbalance leads to cytokine-mediated immune evasion mechanisms that may influence the success of stem cell-derived immunotherapies [87]. They use various strategies to avoid detection and destruction by the immune system, such as secreting immunosuppressive molecules, creating an immunosuppressive TME, and changing their surface markers [88]. The effectiveness of immune cells produced from stem cells can be weakened by cancer cells'frequent development of defense mechanisms against immune detection, such as downregulating immune checkpoints or modifying antigen presentation. For a detailed discussion on how the TME affects therapy, see Sect."Tumor Microenvironment Modulation". Tumor cells use diverse strategies to avoid immune surveillance, including the activation of immune checkpoints, and the induction of immune dysfunction, and the loss or downregulation of antigens. Tumor cells possess the ability to decrease or entirely stop the expression of antigens targeted by immune cells, rendering them undistinguishable to the immune system. This ability allows tumor cells to evade detection and destruction by the body's immune defence [89].

Moreover, cancer cells are able to elevate the level of immune checkpoint molecules such as Cytotoxic T-lymphocyte associated protein 4 (CTLA-4) and programmed death-ligand 1 (PD-1), which help them suppress the immune response [89]. For example, immune checkpoint molecules such as PD-L1 are expressed by CSCs, and they bind to the immune cells'inhibitory receptors to stop them from attacking tumors [90]. Additionally, immune cell function may be hampered by tumor cells'ability to create an immunosuppressive environment in the TME [89]. CSCs can regulate the TME to produce an immunosuppressive environment that suppresses anti-tumor immune responses through the secretion of factors that attract regulatory T cells (Tregs) and myeloid-derived suppressor cells (MDSCs) [90]. These immune evasion strategies make it far more difficult for therapeutic stem cells or the immune cells to find and destroy cancer cells. It appears as though the tumor is continuously constructing defenses and changing its disguise. It is a complicated battle since tumors employ multiple of these strategies simultaneously. Because tumors are so adaptable, it is still very difficult to identify a single, universally effective cure, despite scientists'concerted efforts to inhibit various evasion strategies.

### Regulatory and Manufacturing Barriers

The use of stem cells in cancer treatment is a relatively new field of medicine, and the regulatory frameworks that govern them are still evolving; as a result, this can lead to uncertainty and delays in the development and approval processes [91]. Furthermore, moral concerns related to the utilization of embryonic stem cells have raised controversy and regulatory issues [92]. The Food and Drug Administration (FDA) are an example of the regulatory agency that is responsible for guaranteeing the safety and efficiency of stem cell therapies as it sets standards, evaluates clinical trial data, and grants or denies products for commercial usage [93]. FDA/EMA standards include the need for genomic stability data (e.g., karyotyping, off-target CRISPR analysis) and potency assays (e.g., cytotoxicity for NK cells) for iPSC products [94]. While the EMA demands more stringent potency tests for MSCs, the FDA's RMAT approach expedites clearance for CAR-T but necessitates post-market surveillance. For example, the withdrawal of JCAR015 owing to neurotoxicity [94]. The International Society for Stem Cell Research (ISSCR) has developed guidelines addressing the distinctive challenges and opportunities associated with the usage of stem cells in evolving countries. These guidelines emphasize the necessity for these therapies to be accessible and affordable to all patients [94]. Furthermore, the guidelines emphasize the importance of the safety and well-being of patients in stem cell-based therapy clinical trials and applications, which requires a comprehensive assessment of potential risks and benefits [95].

Stem cell-based therapies also face significant challenges in manufacturing and scaling up, requiring rigorous controls to ensure consistency in quality, safety, and efficacy in large-scale clinical settings [96]. Some of the manufacturing challenges include less than 50% differentiation efficiency for NK cells, and 30% of CAR-T expansion failure rates because of T-cell fatigue [96, 97]. The progress of scalable and effective manufacturing procedures is of paramount significance for the production of considerable amounts of IPSC-based products while preserving the requisite quality and consistency, including the developments in automation technology, cell culture media, and bioreactors [97]. With regard to HSC, although HSC-based therapies hold a lot of potential, there are still many significant challenges that must be overcome, including ensuring the safety of gene-edited cells, developing efficient manufacturing processes, and studying the long-term effects of these treatments [98].

### Translational and Clinical Challenges

Regardless of the breakthroughs in engineered cell therapies, clinical interpretation continues to be obstructed by TME resistance, constrained perseverance, and toxicity. Current clinical trials show that there are still obstacles in translating stem cell therapies to the clinic. Due to stromal barriers, allogeneic CAR-T studies demonstrated less than 10% infiltration efficiency in solid tumors, indicating that TME resistance is still a significant obstacle [99]. Additionally, phase I trials results for iPSC-derived NK cells showed significant cytotoxicity but limited persistence in less than 14 days [100]. Furthermore, immune-related adverse events (irAEs), demonstrate unresolved safety concerns. For example, in anti-CD19 CAR-T studies, 20–30% of patients suffered from severe neurotoxicity. While anti-CD19 CAR-T therapies achieve 80–90% complete remission rates in B-ALL, solid tumors show less than 20% response rates due to stromal barriers and antigen heterogeneity [69]. Recent Phase II trials of GD2-targeted CAR-T for neuroblastoma reported only 28% overall response rate, with a median PFS of 4.2 months.

Scalability is constrained by high costs and strict FDA/EMA regulations. Genomic stability such as off-target CRISPR analysis, and potency assays such as cytotoxicity for NK cells are required for iPSC products[97]. The withdrawal of JCAR015 due to neurotoxicity highlights the necessity of robust post-market surveillance [101]. Furthermore, donor consent, genetic modification, and fair access are ethical issues surrounding iPSC-derived treatments [94].

## Advances in Overcoming Current Challenges

### Gene Editing and CRISPR

CRISPR-Cas9 is a revolutionary gene-editing technology that has opened up new opportunities for enhancing and improving the safety of stem cell-based immunotherapies. Clinical trials initiated in 2025 have explored CRISPR-edited T cells targeting specific negative regulators like CISH, demonstrating improved anti-tumor activity and early signs of durable responses in solid tumors [102].These advancements highlight the potential of precision gene editing to overcome immunotherapy resistance. By precisely modifying immune cell genes derived from stem cells, researchers can enhance their durability, reduce tumorigenicity risks, and improve tumor-targeting capabilities. For instance, CRISPR-Cas9 has been used to edit T cell genes to target specific antigens, resulting in more refined and effective T cell therapies in preclinical and clinical studies. This genome editing technique has enhanced the adaptability of T cells to particular microenvironments, allowing for the advancement of T cell therapies [103]. CRISPR-Cas9 can modify the genes of primary T cells and engineered T cells, such as CAR-T and TCR- T either in vitro (outside the body) or in vivo (within the body) to regulate T cell differentiation and activation states [103]. Editing genetic material enhances T cell functionality for effectively targeting and destroying cancer cells. Moreover, CRISPR-Cas9 can directly alter or disable genes within the cancer that promote tumor growth or survival. M. Azangou-Khyavy et al*.* (2020) state that various clinical experiments are presently being conducted to evaluate the safety and effectiveness of CRISPR-Cas9-based cancer treatments in patients with different cancer types, including leukemia, lymphoma, and solid tumors. Preliminary results from these trials show promising outcomes, including prolonged survival and effective tumor reduction, highlighting the potential of CRISPR-Cas9 in cancer immunotherapy [104].

### Targeted Drug Delivery Systems

**Fig. 9:**
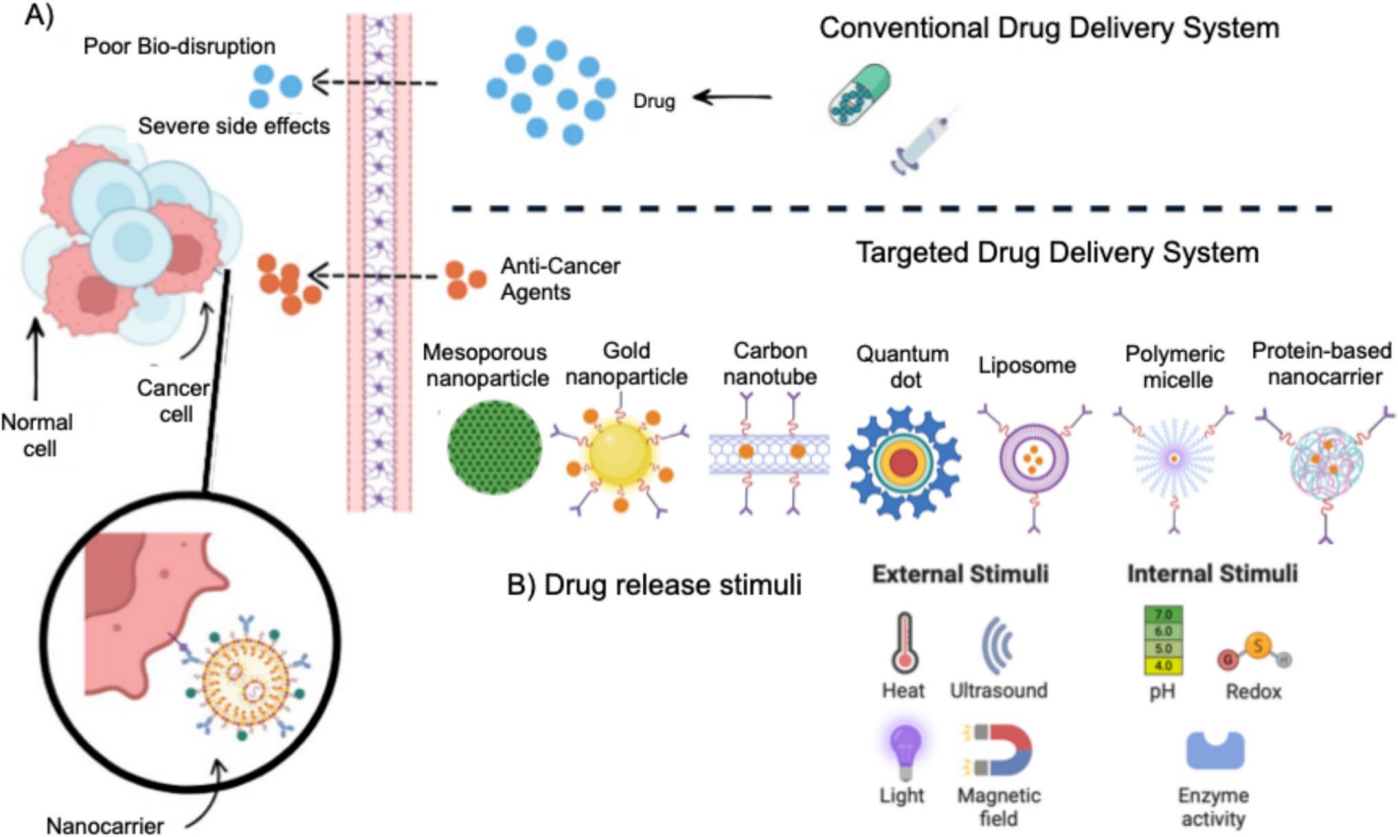
A) The difference between conventional drug delivery systems and targeted drug delivery systems. Conventional drug delivery system has poor bio-disruption and severe side effects. Targeted drug delivery system shows better results as anti-cancer agents interact and eliminate cancer cells. B) External and internal drug release stimuli (Created with BioRender)

Researchers are actively developing new drug delivery systems using various materials, such as nanocarriers, nanoparticles, and nanofilms, which are typically biocompatible and biodegradable. They can also be designed to have a longer circulation time in the bloodstream, thereby facilitating sustained drug delivery [105]. These new drug delivery system formulations offer several advantages over traditional methods, including increased treatment efficacy, reduced adverse side effects, and controlled drug release [105, 106]. Studies have shown that targeted drug delivery is a precise and effective strategy in oncology, where drugs are delivered precisely to tumor cells or tissues, thereby enhancing their treatment efficacy while simultaneously minimizing the unwanted side effects on healthy cells or tissues [107]. Developments in targeted drug delivery systems, including the use of nanoparticles or biomaterial scaffolds, have enhanced the accuracy of stem cell therapies in reaching tumor sites while reducing off-target effects and optimizing therapeutic benefits. Figure 9 shows the difference between conventional drug delivery systems and targeted drug delivery systems.

K. Tang et al*.* (2012) demonstrate evidence that microparticles, which contain chemotherapy drugs, are capable of efficiently killing other tumor cells, which raises the possibility of a potential therapeutic strategy whereby the body’s own tumor cells serve as delivery vehicles for targeted drug delivery [108]. MSCs are naturally occurring stem cells that possess the capacity to develop into multiple types of cells. They can also be genetically modified to produce therapeutic proteins or loaded with drugs, making them ideal carriers for targeted drug delivery. This strategy may prove to be a useful tool for the treatment of a variety of illnesses, including cancer [109]. Extracellular vesicles derived from MSCs, such as exosomes, offer several benefits as drug delivery carriers, including being capable of targeting specific tissues, a low likelihood of inducing an immune response, and the ability to transport a variety of therapeutic agents [110].

Targeted drug delivery systems still face challenges. One significant issue is ensuring that the medications only reach the tumor and nowhere else. Thick tumor tissue or quick immune system clearance might also prevent specific delivery methods from reaching their intended location. Even if they succeed, scientists are still trying to figure out how to ensure that the medication is released into the cancer cells at the appropriate time and quantity.

### Enhanced Stem Cell Engineering

CAR T cells are immune cells that have been genetically modified to identify and attack cancer cells. Using CRISPR-Cas9 technology, one extremely promising area of cancer immunotherapy is the modification of T cells, a subset of immune cells that are essential for recognizing and destroying cancer cells. CRISPR/Cas9 technology can be used to improve the effectiveness of CAR-T cells and TCR-T cells with a more remarkable ability to fight tumors [111]. For example, CAR-T cells possess genetically modified receptors that allow them to identify particular antigens found on cancer cell surfaces, ultimately destroying them. Similarly, researchers can genetically modify TCR-T cells to identify and target antigens found on cancer cells, enhancing their capacity to precisely identify and eliminate cancer cells [112]. These genetically modified TCR-T cells can precisely target and destroy cancer cells because they produce TCR receptors that are capable of recognizing and detecting tumor-specific antigens [113]. The CRISPR/Cas9 method can also be used to insert CARs into T cells, generating CAR-T cells. These modified receptors are engineered to enable T cells to detect and destroy cancer cells [114]. Moreover, CRISPR/Cas9 can be utilized to deactivate genes that limit the effectiveness of CAR-T cells, such as PD-1 and CTLA-4 [114].

**Fig. 10:**
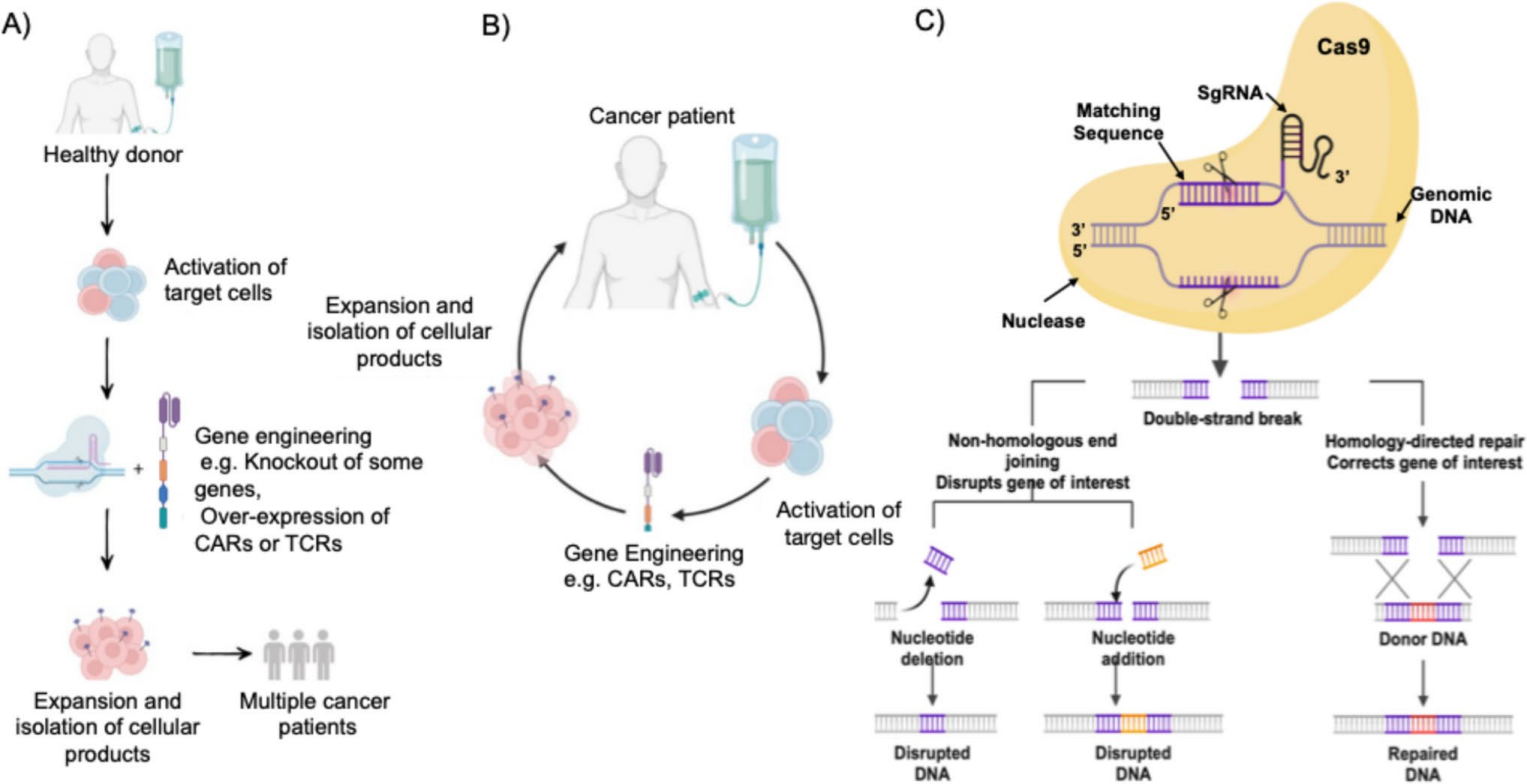
Stem cell engineering process. A) Engineering stem cells from a healthy donor: Target cells are activated, followed by expansion and isolation of cellular products. These cells then undergo Gene engineering, which can involve Knockout of some genes or Over-expression of Chimeric Antigen Receptors (CARs) or T-Cell Receptors (TCRs). B) Engineering stem cells from the patient. C) Gene editing using CRISPR-Cas9 where A single guide RNA (sgRNA) with a Matching Sequence guides the Cas9 protein to a specific site in the Genomic DNA. (Created with BioRender)

Furthermore, researchers are developing new ways to design and engineer CAR-NK cells to improve their effectiveness and targeting, including the fusion of additional signaling molecules to CAR receptors, which is aimed at enhancing NK cells’ capacity to distinguish and terminate cancer cells [115]. Employing bioengineered MSCs in cancer treatment represents a tailored approach that minimizes damage to healthy tissues. MSCs can travel to places that have tumor, making them effective delivery carriers of therapeutic agents such as drugs or genes [116]. Moreover, their immunomodulatory properties can help in overcoming tumor-induced immunosuppression, strengthening the body's natural defenses against cancer [116]. Figure 10 shows the stem cell engineering process from healthy donor as well as cancer patient, and gene editing using CRISPR-Cas9. The Cas9 enzyme induces a double-strand break (DSB) at the specific target site, which is subsequently repaired by the cell's natural repair mechanisms. In Non-Homologous End Joining (NHEJ), broken DNA ends are joined together, but this process often introduces errors such as insertions or deletions. In contrast, Homology-Directed Repair (HDR) utilizes a donor DNA template to accurately repair the break, enabling precise edits, such as correcting a gene or inserting new sequences, to produce repaired DNA. However, even with these encouraging advancements, challenges still exist. For example, changing stem cells so much can make it difficult for them to survive and function properly once inside the body. There's also a risk that substantial engineering could unintentionally alter cell behavior unexpectedly. Furthermore, engineering specialized stem cells for each patient can be costly and complex, making it challenging to provide personalized treatments widely.

## Future Directions and Emerging Trends

The future of stem cell-based immunotherapy lies in developing allogeneic therapies, personalized stem cell approaches, and combination therapy.

### Allogeneic Stem Cell Therapies

Natural universal donor T cells can be employed for “Off-the-shelf” allogeneic T cell treatment for cancer [117]. These treatments are designed to be readily available and effective for all patients, irrespective of their human leukocyte antigen (HLA) type. γδ-TCR T cells possess a distinctive T cell receptor (TCR) that enables them to recognize a diverse array of antigens, including those associated with tumors, making them helpful in targeting cancer cells that express various antigens [117, 118]. iPSCs can be genetically modified to increase their therapeutic potential, and differentiate into multiple cell types, including T cells. The progress of"off-the-shelf"therapies derived from T cells generated by iPSC is a promising step in cancer treatment, providing a treatment option that can be scaled up to be readily available, contributing to a readily available and scalable therapeutic alternative [119]. However, a major issue with allogeneic stem cell therapies is ensuring that the patient's immune system does not reject the donor cells while maintaining their high potential to combat cancer.

### Personalized Stem Cell-Based Cancer Immunotherapy

The employment of iPSCs to produce a variety of cell types, such as dendritic cells and macrophages, for cancer immunotherapy has demonstrated future potential in enhancing immune activation and reducing rejection, customized cancer vaccines, large-scale production, and cost-effectiveness [37]. Furthermore, the utilization of iPSCs obtained from a patient's cells represents a promising approach for personalized therapies. This approach enables the potential production of immune cells that precisely align with the immune system of the patient and are customized to target the unique antigens of their tumors more successfully. Due to their scalability and genetic modification potential, NK cells obtained from IPSCs represent a viable avenue for cancer immunotherapy. These stem cells can generate NK cells in large quantities, bypassing the limitations associated with traditional NK cell sources [120]. In addition, specific receptors can be genetically modified to improve their capacity to recognize and eliminate tumor cells. Pre-clinical studies have shown that NK cells derived from iPSCs are effective in eliminating cancer cells, which makes them a promising option for the development of personalized cancer immunotherapies [120]. NK cells constitute a key component of the innate immune system, exhibiting an essential function in the combat against cancerous cells due to their distinctive capacity to identify, target, and eliminate aberrant cells without the necessity of prior stimulation or immunization [121]. In recent years, immunotherapies based on NK cells have witnessed remarkable development as genetic engineering techniques have been developed to improve the functions of these cells and enhance their ability to attack cancer cells more effectively and specifically. These treatments show positive results in many clinical trials and are a safe and effective therapeutic option for combating malignant tumors [121].

Furthermore, M. Kishi et al*.* (2021) discuss the potential of iPSC-based vaccines to target CSCs, a subset of cancer cells with high self-renewal capacity and resistant to conventional therapies. The results show that iPSC-based vaccines can induce a robust immune response against CSCs, reducing tumor size and metastasis. The research also elucidates the potential mechanism behind these effects, which involves the activation of cytotoxic T cells (CTLs) and NK cells [122]. These findings are promising for cancer treatment, as they suggest the potential for developing new and effective therapies targeting CSCs. However, further studies are needed to evaluate the safety and efficacy of these vaccines in human clinical trials. Additionally, the process of creating personalized immunotherapies is time-consuming and complex and frequently quite costly. One significant practical obstacle that must be addressed is scaling up manufacturing so that everyone using them can do so.

### Combination therapies

The latest research indicates a growing interest in combining stem cell therapies with next-generation immunotherapies, including bispecific antibodies, immune-activating vaccines, and traditional treatments such as radiation and chemotherapy. Combining stem cell-based therapies with other treatments is anticipated to improve cancer therapy’s accuracy and effectiveness even more. Study findings indicate that immunotherapy can be combined with additional treatments to increase therapeutic efficacy and prolong patient survival time significantly [123]. Another study has shown that the administration of neoadjuvant personalized cancer vaccines to cancer patients prior to the primary treatment represents a promising new approach to cancer treatment, as it shrinks tumors and makes them easier to remove by stimulating the immune system of the patient to fight cancer [124]. Other studies suggest that using bispecific antibodies in combination with other cancer therapies can be more effective in treating cancer and reduce the risk of resistance as it is designed to detect EGFR and EPHA2 proteins, which are located on the cancer cells’ surface [125, 126]. Bispecific antibodies are a specific class of antibodies capable of binding to two distinct antigens simultaneously. This distinctive attribute enables them to regulate the immune system in ways that are not feasible with traditional monoclonal antibodies. Consequently, bispecific antibodies hold promise to be utilized in treating a wide range of illness, including cancer [127]. Developing bispecific antibodies has improved the idea of utilizing the T cells of a cancer patient to eliminate and fight cancerous cells [128].

Moreover, ultrasound is a promising technology in cancer therapy, as it can be used to stimulate an anti-tumor immune response. The main biological effects of ultrasound include the thermal effect to heat and kill cancerous tissue, the mechanical effect to facilitate the entry of drugs and immunomodulators into cancer cells, the photochemical effect to produce reactive oxygen species that can kill cancer cells, and the electroacoustic effect to stimulate the release of cytokines and activate immune cells [129]. Nanomaterials can be used to enhance the effect of ultrasound in cancer immunotherapy in several ways, including improving the delivery of drugs and immunomodulators to cancer tissues, increasing the absorption of ultrasound energy, and providing additional functions, such as imaging and targeting [129].

Determining the optimal combination of treatments, including which ones to use, in what quantity, and in what order, is a difficult analytical challenge. Adding medicines to the mix may result in side effects, which require close monitoring in studies. Running clinical trials for combined therapies is, therefore, far more difficult, requiring careful planning to understand how they work together.

## Conclusion

Stem cell-based immunotherapy is considered a rapidly developing, promising field in cancer treatment because it provides a promising alternative to traditional therapies such as radiation therapy and chemotherapy. Nevertheless, several challenges interfere with using this technology, including safety concerns regarding the ability of stem cells to promote cancer growth and the regulatory and manufacturing challenges that limit the application of this therapy. Recent advances have helped enhance these treatments’ effectiveness, including the development of gene editing using CRISPR and targeted drug delivery systems. Several studies indicate that combining stem cell immunotherapy with other therapies might improve their efficiency and prolong the survival of cancer patients. This field is expected to provide effective treatment solutions for several patients with cancer shortly through the expansion of personalized therapies.

## Data Availability

All of the data is presented within this article.
